# Knowledge of glaucoma and associated factors among primary glaucoma patients in Kunming, China

**DOI:** 10.1186/s12886-022-02322-0

**Published:** 2022-02-28

**Authors:** Xi Chen, Yun-Long Zhong, Qin Chen, Yi-Jin Tao, Wen-Yan Yang, Zhi-Qiang Niu, Hua Zhong, Qing Cun

**Affiliations:** 1 Department of Ophthalmology, the First Affiliated Hospital of Kunming Medical University, Kunming, 650032 Yunnan Province China; 2 Department of Ophthalmology, the First Affiliated Hospital of Nanjing Medical University, Nanjing, 210029 Jiangsu Province China

**Keywords:** Knowledge, Glaucoma patients, Associated factors, Medication compliance, Glaucoma-related quality of life

## Abstract

**Background:**

To investigate the level of knowledge, attitude, and practices about glaucoma and associated factors among primary glaucoma patients in Kunming, China.

**Methods:**

A hospital-based study was conducted on 93 patients from the First Affiliated Hospital of Kunming Medical University. Interviewer-administered questionnaires were used to collect data after written informed consent. Data were analyzed by SPSS 19.0. Univariate and multivariate logistic regression models were used to identify factors. A Chi-square test was used to analyze the association between knowledge of glaucoma and medication compliance, Mann–Whitney U test was performed to assess the relationship between knowledge of glaucoma and quality of life in patients with glaucoma. *P*-value < 0.05 was considered statistically significant.

**Results:**

Among 93 patients**,** 55 (59.14%) were aware of glaucoma, 48 (51.61%) had good knowledge of glaucoma, while 45 (48.39%) had poor knowledge. Younger age and duration of glaucoma were associated positively with knowledge of glaucoma. 87 (93.54%) patients got knowledge of their disease from doctors. 79.17% of respondents could use all the anti-glaucoma medications on time, out of which 54.17% had good knowledge of glaucoma while 25.00% had poor knowledge of glaucoma. 30.56% of respondents used to stop anti-glaucoma medications on their own out of which only 9.72% had good knowledge of glaucoma while 20.83% had poor knowledge of glaucoma. Patients with good knowledge of glaucoma had lower scores on the Glaucoma Quality of Life-15 questionnaire. Thus, the compliance to anti-glaucoma medications and glaucoma-related quality of life were better in patients with good knowledge.

**Conclusions:**

The level of knowledge of glaucoma among patients in Kunming is relatively low. Improving knowledge with suitable content for patients through effective multiple means such as the mass media rather than relying only on ophthalmologists may be a veritable first step in combating blindness from glaucoma and enhancing patients' quality of life.

**Supplementary Information:**

The online version contains supplementary material available at 10.1186/s12886-022-02322-0.

## Background

Glaucoma is a major eye health problem that leads to irreversible visual impairment. The global number of people with glaucoma is expected to increase to 111.8 million in 2040 [1]. East Asia and South-Central Asia will harbour the highest number of glaucoma cases worldwide [2]. As the lack of remarkable symptoms in early stage generally, it is difficult to be aware of the vision loss for glaucoma patients until the severe visual impairments occur. Increased knowledge of glaucoma may alert patients regarding subtle symptoms and lead them to seek appropriate medical care at an earlier time. Early detection is crucial, which may prevent blindness from glaucoma. Glaucoma is a chronic, progressive disease and most patients of glaucoma require a long-term or even lifelong management and follow-up. It is key for patients to have good compliance to the long-term management, including treatment adherence and regular follow-up [3]. Previous reports showed that the compliance to the management of glaucoma was better in patients with good knowledge of their disease [4–6]. Thus, raising public knowledge of glaucoma, particularly for patients, plays an important role in glaucoma early detection and treatment.

The knowledge of glaucoma has not been accurately defined. It is widely accepted that respondents "having heard of glaucoma" even before they were contacted/recruited for the study were defined as "aware," and respondents having who defined some understanding of the eye disease as "knowledgeable" [7, 8]. Many studies on the knowledge of glaucoma across different countries indicate that glaucoma knowledge is still low, especially in developing countries [7–10]. Even in developed countries, patients remain unfamiliar with key aspects of their disease [5, 6, 11]. Published studies worldwide regarding knowledge of glaucoma bring to light the need to intensify efforts to improve the knowledge [12, 13]. Increasing knowledge of glaucoma and creating awareness about the importance of early diagnosis and treatment in preventing blindness is the key to glaucoma control [14].

China is the world’s most populous country and may have the greatest glaucoma burden [15]. The number of people with all glaucoma in China was 13.12 million in 2015. By 2050, the number of all glaucoma cases in China will be 25.16 million [16]. Assessment of knowledge level toward glaucoma in China is a critical step in preventing blindness. Existing data on the knowledge of glaucoma in China showed that China's general population lacks knowledge about glaucoma [17, 18]. In the Hong Kong Chinese population, only 10.2% of participants could describe glaucoma symptoms correctly [17]. A Focus Group Study in rural China showed that physicians and patients understood glaucoma only as an acutely symptomatic disease of relatively low prevalence [19]. However, few studies have reported factors that influencing the knowledge level in China, especially for glaucoma patients.

Therefore, we conducted this cross-sectional study using a hospital-based design to assess the related level of knowledge, attitude, and practices (KAP) about glaucoma and associated factors among patients in Kunming, an important central city in southwest China.

## Methods

### Study design

This was a hospital-based, cross-sectional study. The study was approved by the hospital's ethics committee and was conducted according to the tenets of the Declaration of Helsinki. All participants gave their written informed consent. The data were labeled with serial numbers and deidentified prior to analysis. No personal identifiers were obtained.

### Study population and participant selection

This study enrolled patients with clinically diagnosed primary glaucoma scheduled for vision care at the First Affiliated Hospital of Kunming Medical University, a public hospital in the southwest city of China, from June 2013 to November 2013. The patients were followed for 4 years. The inclusion criteria were Chinese-speaking adult patients (18 years old and above) with a primary glaucoma diagnosis. This study included patients with primary open angle glaucoma (POAG) and primary angle-closure glaucoma (PACG). In this study, diagnosis of POAG was based on the following abnormalities: (1) evidence of optic nerve damage presented as a structural abnormality of the optic disc or retinal nerve fiber layer; (2) characteristic abnormalities in the visual field; (3) a normal appearing open anterior chamber angle and absence of secondary causes of glaucoma; (4) Intraocular pressure (IOP) > 21 mmHg (no IOP lowering therapy). Juvenile open-angle glaucoma (JOAG) was diagnosed when patients’ age at the time of POAG diagnosis was younger than 35 years. Diagnosis of PACG was based on the following abnormalities: (1) evidence of glaucomatous optic neuropathy; (2) characteristic visual field defect; (3) angle closure; (4) IOP > 21 mmHg (no IOP lowering therapy). The exclusion criteria were as follows: (1) secondary glaucoma; (2) any other coexisting ocular condition that could impair visual function (e.g., corneal opacity, cataract more than N2, C2 or P2 according to the Lens Opacities Classification System II, retinal pathology, optic neuropathy, other than glaucoma); (3) disability in a visual field test due to cognitive impairment.

### Clinical assessment

All patients underwent a complete ophthalmologic examination, including best-corrected visual acuity (BCVA), slit-lamp biomicroscopy, tonometry, gonioscopy, funduscopy, and visual field examination [20]. BCVA was evaluated with Snellen equivalents, based on a standard refraction and testing protocol at a starting distance of 5 m [20]. IOP was measured using a Goldmann applanation tonometer (AT-900, HAAG-STREIT, Switzerland) [20]. Visual field was performed with automated perimetry (Humphrey750i, Zeiss, Germany) using a 24–2 threshold program (HFA24-2) with the SITA-Fast strategy.

Demographic information including gender, age, ethnicity, occupation, educational level, income, and travel distance to hospital, together with clinical information including duration of glaucoma, family history of glaucoma, medication treatment, surgical treatment, and type of glaucoma were all recorded on a checklist [20].

Two questionnaires were administered to all subjects: a KAP of glaucoma questionnaire and a Glaucoma Quality of Life-15 questionnaire (GQL-15) [21, 22]. One research staff interviewed each patient face to face and recorded demographic information, clinical information, and two questionnaires.

### Evaluation and operational definitions of knowledge of glaucoma

The KAP of glaucoma questionnaire was designed using previous studies from the literature [7, 8, 23–27] and modified according to Chinese lifestyle and habits. The questionnaire contained 20 questions assessing knowledge, attitude, and practices about glaucoma: the knowledge section contained 7 questions (Questions1-7), the attitude section contained 5 questions (Questions 8–12), and the practices section contained 8 questions (Questions 13–20). Answers of knowledge section questions were graded, and scores of questions 1–7 were summed up to assess glaucoma knowledge. Higher scores meant better knowledge of glaucoma. The questionnaire content and the scoring scheme are displayed in Table 1.

**Table 1:** Content of KAP of glaucoma questionnaire and scoring scheme

Questions	Scores
1. Do you know what kind of eye disease you have?	Yes: 1; No: 0
2^a^. What is glaucoma?	Satisfactory:1; Unsatisfactory:0
3. Can glaucoma be cured without visiting doctors?	Yes: 0; No: 1
4^a^. What is normal intraocular pressure?	Satisfactory:1; Unsatisfactory:0
5. Does glaucoma cause blindness?	Yes: 1; No: 0
6^a^. Why is the visual field measured?	Satisfactory:1; Unsatisfactory:0
7. Is it important to have glaucoma screening for your relatives?	Yes: 1; No: 0
8. Do you know your intraocular pressure at your last visit?	
9. When did you first know you had glaucoma?	
10. Did you have any difficulty with your vision when you first knew you had glaucoma?	
11. What kind of difficulty did you have with your vision?	
(if ‘yes’ for above)	
12. What were your sources of information about glaucoma?	
13. Have you ever used anti-glaucoma medications (pills or eyedrops)?	
14. What is(are) your medication(s)? How many times per day do you	
use it (them)? (if ‘yes’ for above)	
15. Can you use all the anti-glaucoma medications on time?	
16. Have you ever stopped the medications on your own?	
17. Which medication(s) did you stop? (if ‘yes’ for above)	
18. Do the anti-glaucoma medications have side effects?	
19. Have the side effects of the medications been explained to you?	
20^a^. What is the purpose of the medications you use?	

KAP knowledge, attitude, and practices

^a^Satisfactory answers for questions 2, 4, 6, and 20 are as follows:

2: The disease is caused by increased intraocular pressure or the disease can affect the optic nerve or the disease can affect the visual field;

4: Any numbers in the range 10-21 mmHg;

6: Optic nerve damage can cause visual field damage or visual field can be used to analyze the progress of glaucoma;

20: Reducing intraocular pressure or preventing the development of glaucoma;

Answers other than satisfactory answers are considered to be unsatisfactory answers

A participant was classified as aware of glaucoma if a satisfactory response was obtained to the question ‘What is glaucoma?’. In this study, hearing glaucoma alone was not considered as knowledge because merely being aware of the term did not ensure knowledge about the disease among glaucoma patients.

Respondents who scored above or equal to the median score (6[4^~^7]) were considered to have good knowledge, while those who scored below the median score were considered to have poor knowledge.

### Glaucoma quality of life-15 questionnaire

Specifically designed to assess the quality of life (QoL) in patients with glaucoma, GQL-15 has been shown to correlate strongly with visual disability and psychophysical measures of visual function and have a high test–retest reproducibility. Higher scores of GQL-15 questionnaires represented poorer glaucoma-related QoL (G-QoL) [21, 22]. Thus, this study used the Chinese version of GQL-15 to explore the association between knowledge of glaucoma and G-QoL.

### Data analysis

SPSS 19.0 statistics software (IBM Corporation, Armonk, NY, USA) was used for statistical analysis. Crude relationships between demographic and clinical variables and glaucoma knowledge were explored with univariate logistic analysis. The remaining demographic and clinical variables then were subjected to a forward stepwise selection procedure in which variables significantly associated with the outcome at the *p* < 0.1 level were included. To examine the independent effects of demographic and clinical variables on glaucoma knowledge, we constructed multivariate logistic regression models. Adjusted odds ratios and the associated 95% confidence intervals for attributes that are independent predictors of knowledge of glaucoma at the *p* < 0.05 level were reported. A Chi-square test was used to analyze the association between knowledge of glaucoma and medication compliance, Mann–Whitney U test was performed to assess the relationship between knowledge of glaucoma and G-QoL. A p-value of < 0.05 was considered statistically significant.

## Results

### Subjects' characteristics

Ninety-three patients with primary glaucoma were chosen as study participants. The sample consisted of 43 males (46.24%) and 50 females (53.76%). The mean age of the included subjects was 55.62 ± 16.70 years (range: 20–91 years). 54 patients had POAG/JOAG, and 39 patients had PACG. Table 2 shows the demographic and clinical characteristics of these subjects and median scores of glaucoma knowledge.

**Table 2:** Sociodemographic, clinical characteristics of subjects and median scores of knowledge of glaucoma

Variable	Frequency (Percent %)	Median score
Gender		
Male	43 (46.24%)	5 (4^~^7)
Female	50 (53.76%)	6 (4^~^7)
Age(years)		
≥ 60	46 (49.46)	4 (3^~^6)
40 to < 60	24 (25.81)	6 (5^~^7)
18 to < 40	23 (24.73)	7 (5^~^7)
Ethnicity		
Han Chinese	80 (86.02%)	6 (4^~^7)
Ethnic minorities	13 (13.98%)	5 (3.5^~^6.5)
Occupation		
Farmer	26 (27.96%)	4 (3^~^4.25)
Worker	31 (33.33%)	5 (4^~^6)
Skilled/Professional^a^	36 (38.71%)	7 (6^~^7)
Educational level		
Illiteracy and Primary school	31 (33.33%)	4 (3^~^4)
Middle school	18 (19.35%)	5.5 (4^~^7)
High school	18 (19.35%)	6 (5.75^~^7)
College and above	26 (27.96%	7 (6^~^7)
Income (Yuan /month)		
< 1000	34 (36.56%)	4 (3^~^4.25)
1000 to 3000	42 (45.16%)	6 (5^~^7)
> 3000	17 (18.28%)	7 (6^~^7)
Duration of glaucoma(years)		
< 1	38 (40.86%)	4 (3^~^6)
1 to 5	37 (39.78%)	6 (4^~^7)
> 5	18 (19.35%)	7 (5.75^~^7)
Family history of glaucoma		
No	78 (83.87%)	5.5 (4^~^7)
Yes	15 (16.13%)	6 (5^~^7)
Surgical treatment		
No	36 (38.71%)	6 (4^~^7)
Yes	57 (61.29%)	5 (3.5^~^6.5)
Medication treatment		
No	24 (25.81%)	4 (3^~^6)
Yes	69 (74.19%)	6 (4^~^7)
Travel distance to hospital (km)		
< 100	53 (56.99%)	4 (3^~^6)
100–300	22 (23.66%)	6 (4^~^7)
> 300	18 (19.35%)	6 (4^~^7)
Type of glaucoma		
Primary angle-closure glaucoma	39 (41.94%)	4 (3^~^6)
Primary open angle glaucoma/Juvenile open-angle glaucoma	54 (58.06%)	6 (4^~^7)

^a^Including doctors, nurses, accountants, drivers, and teachers

### Knowledge, and responses to the questions regarding knowledge of glaucoma

**Fig.1:**
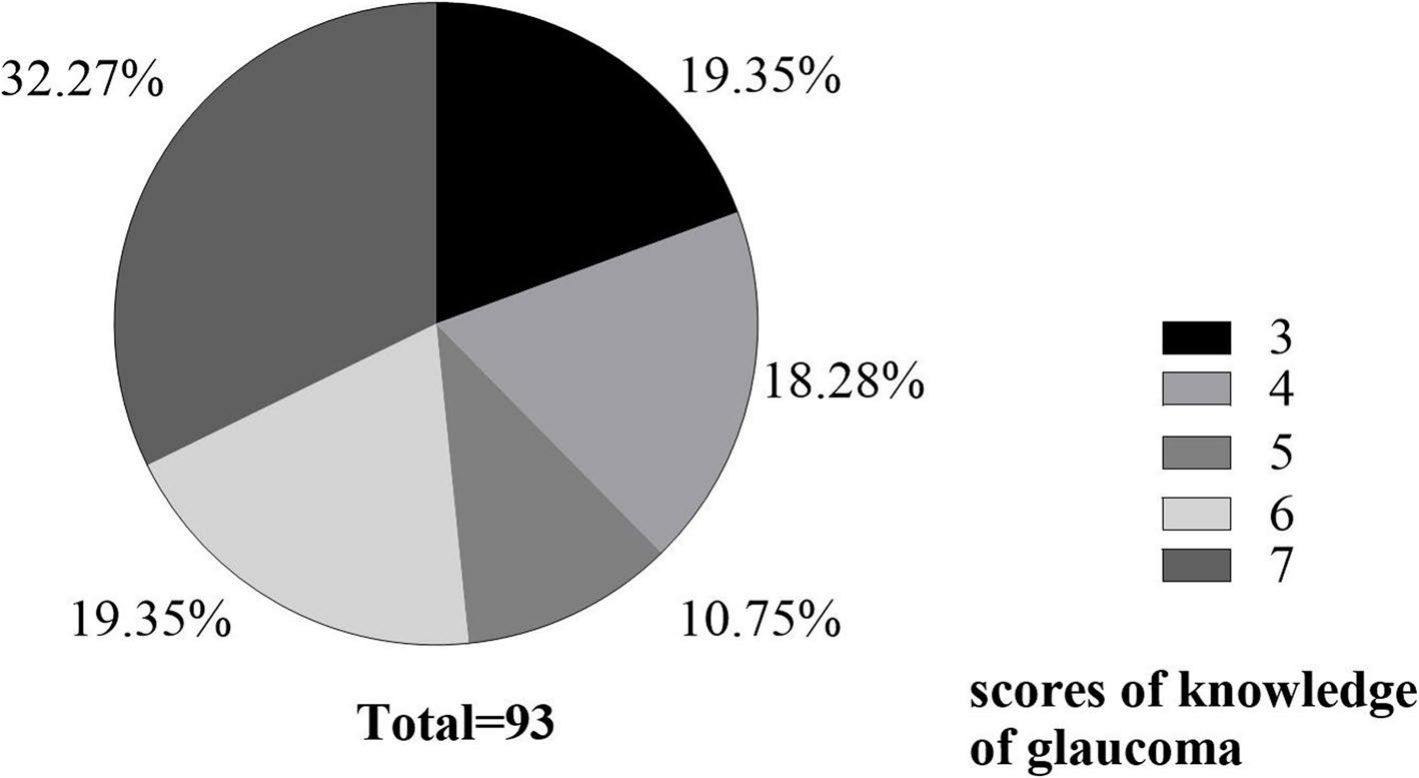
Distribution of glaucoma knowledge scores

Among 93 participants, 98.92% knew what kind of eye disease they had. On the other hand, only 59.14% of them were aware of glaucoma according to our definitions. The scores of our participants ranged from 3 to 7 for the knowledge of glaucoma. Figure 1 summarizes the distribution of scores. Although 32.27% of participants got scores of 7, knowledge of their diseases was still inadequate among glaucoma patients in our survey, especially for those critical questions about the occurrence and development of glaucoma. As shown in Table 3, only 59.14% of patients know what glaucoma and normal intraocular pressure was, 38.71% of patients understood the reason for examining the visual field merely.

**Table 3:** Responses to the questions regarding knowledge of glaucoma

Questions regarding knowledge of glaucoma	Percentage (%)	
1. Do you know what kind of eye disease you have?	Yes (98.92)	No (1.08)
2. What is glaucoma?	Satisfactory (59.14)	Unsatisfactory (40.86)
3. Can glaucoma be cured without visiting doctors?	Yes (2.15)	No (97.85)
4. What is normal intraocular pressure?	Satisfactory (59.14)	Unsatisfactory (40.86)
5. Does glaucoma cause blindness?	Yes (98.92)	No (1.08)
6. Why is the visual field measured?	Satisfactory (38.71)	Unsatisfactory (61.29)
7. Is it important to have glaucoma screening for your relatives?	Yes (74.19)	No (25.81)

### Factors associated with knowledge of glaucoma

Among 93 patients, 48 (51.61%) had good knowledge about glaucoma, while 45 (48.39%) had poor knowledge. The median score was 6(4^~^7).

A logistic regression model evaluated the association of demographic and clinical factors with glaucoma knowledge, and the results were presented in Table 4. The univariate logistic analysis revealed that age (*P* = 0.005, 60 years and older vs. 18–40 years), occupation (*P* = 0.035, farmer vs. worker; *P* < 0.001, farmer vs. skilled/professional), educational level (*P* = 0.004, illiteracy and primary school vs. middle school; *P* < 0.001, illiteracy and primary school vs. high school; *P* < 0.001, illiteracy and primary school vs. college and above), income (*P* < 0.001, less than 1000 yuan/month vs. 1000 to 3000 yuan/month; *P* < 0.001, less than 1000 yuan/month vs. more than 3000 yuan/month), duration of glaucoma (*P* = 0.005, less than 1 year vs. 1–5 years; *P* = 0.001, less than 1 year vs. more than 5 years), medication treatment (*P* = 0.013), travel distance to hospital (*P* = 0.040, 0 to100 km vs. more than 300 km), and type of glaucoma (*P* = 0.033) were significantly associated with glaucoma knowledge (*P* < 0.05). The variables with a *P* value less than 0.10 after the univariate analysis subsequently were included in the multivariate analysis. After adjusting for potential confounders in the multivariate analysis, only younger age (AOR, 10.71; 95% CI, 1.31–87.63; *P* = 0.027; 60 years and older vs. 40–60 years; AOR, 22.50; 95% CI, 1.53–330.96; *P* = 0.023; 60 years and older vs. 18–40 years) and duration of glaucoma (AOR, 22.01; 95% CI, 2.08–232.42; *P* = 0.01; less than 1 year vs. 1–5 years; AOR,107.06; 95% CI, 4.96–2309.92; *P* = 0.003; less than 1 year vs. more than 5 years) were positively associated with knowledge level of glaucoma.

**Table 4:** Univariate and multivariate logistic regression of factor affecting knowledge level of glaucoma

Variable	Knowledge level of glaucoma		COR (95%)	*P*	AOR (95%)	*P*
	**Good (%)**	**Poor (%)**				
Gender						
Male	24(25.81)	19(20.43)	1			
Female	24(25.81)	26(27.94)	0.73(0.32,1.66)	0.450		
Age(years)						
≥ 60	17(18.28)	29(31.18)	1		1	
40 to < 60	14(15.05)	10(10.75)	2.39(0.87,6.55)	0.091	10.71(1.31,87.63)	0.027
18 to < 40	17(18.28)	6(6.45)	4.83(1.60,14.62)	0.005	22.50(1.53,330.96)	0.023
Ethnicity						
Han Chinese	43(46.24)	37(39.78)	1			
Ethnic minorities	5(5.38)	8(8.60)	0.54(0.16,1.79)	0.310		
Occupation						
Farmer	4(4.30)	22(0.24)	1		1	
Worker	13(13.98)	18(19.35)	3.97(1.10,14.32)	0.035	1.45(0.12,17.83)	0.772
Skilled/professional	31(33.33)	5(5.38)	34.10(8.21,141.61)	< 0.001	22.30(0.43,1164.84)	0.124
Educational level						
Illiteracy and Primary school	3(3.23)	28(30.11)	1		1	
Middle school	9(9.68)	9(9.68)	9.33(2.07,42.13)	0.004	0.88(0.06,12.75)	0.922
High school	14(15.05)	4(4.30)	32.67(6.41,166.50)	< 0.001	5.19(0.29,92.63)	0.263
College and above	22(23.66)	4(4.30)	51.33(10.39,253.67)	< 0.001	0.66(0.01,61.72)	0.857
Income (yuan/month)						
< 1000	5(53.76)	29(31.18)	1		1	
1000 to 3000	29(31.18)	13(13.98)	12.94(4.09,40.97)	< 0.001	6.28(0.25,159.35)	0.265
> 3000	14(15.05)	3(3.23)	27.07(5.65,129.72)	< 0.001	2.04(0.04,95.81)	0.717
Duration of glaucoma (years)						
< 1	11(11.83)	27(29.03)	1			
1 to 5	23(24.73)	14(15.05)	4.03(1.54,10.59)	0.005	22.01(2.08,232.42)	0.010
> 5	14(15.05)	4(4.30)	8.59(2.31,31.96)	0.001	107.06(4.96,2309.92)	0.003
Family history of glaucoma						
No	39(41.94)	39(41.94)	1			
Yes	9(9.68)	6(6.45)	1.50(0.49,4.62)	0.480		
Surgical treatment						
No	22(23.66)	14(15.05)	1			
Yes	26(27.96)	31(33.33)	0.53(0.23,1.25)	0.147		
Medication treatment						
No	7(7.53)	17(18.28)	1		1	
Yes	41(44.09)	28(30.11)	3.56(1.31,9.69)	0.013	1.17(0.19,7.37)	0.868
Travel distance to hospital (km)						
0 to100	30(32.26)	23(24.73)	1			
> 100 to 300	13(13.98)	9(9.68)	1.11(0.40,3.04)	0.843	2.15(0.28,16.37)	0.459
> 300	5(5.38)	13(13.98)	0.30(0.09,.095)	0.040	0.79(0.09,7.46)	0.840
Type of glaucoma						
PACG	15(16.13)	24(25.81)	1		1	
POAG/JOAG	33(35.48)	21(22.58)	2.51(1.08,5.86)	0.033	0.77(0.13,4.59)	0.774

*COR* crude odds ratio, *AOR* adjusted odds ratio, *CI* confidence interval, *PACG* primary angle-closure glaucoma, *POAG* primary open angle glaucoma, *JOAG* juvenile open-angle glaucoma

### Sources of knowledge about glaucoma

**Fig.2:**
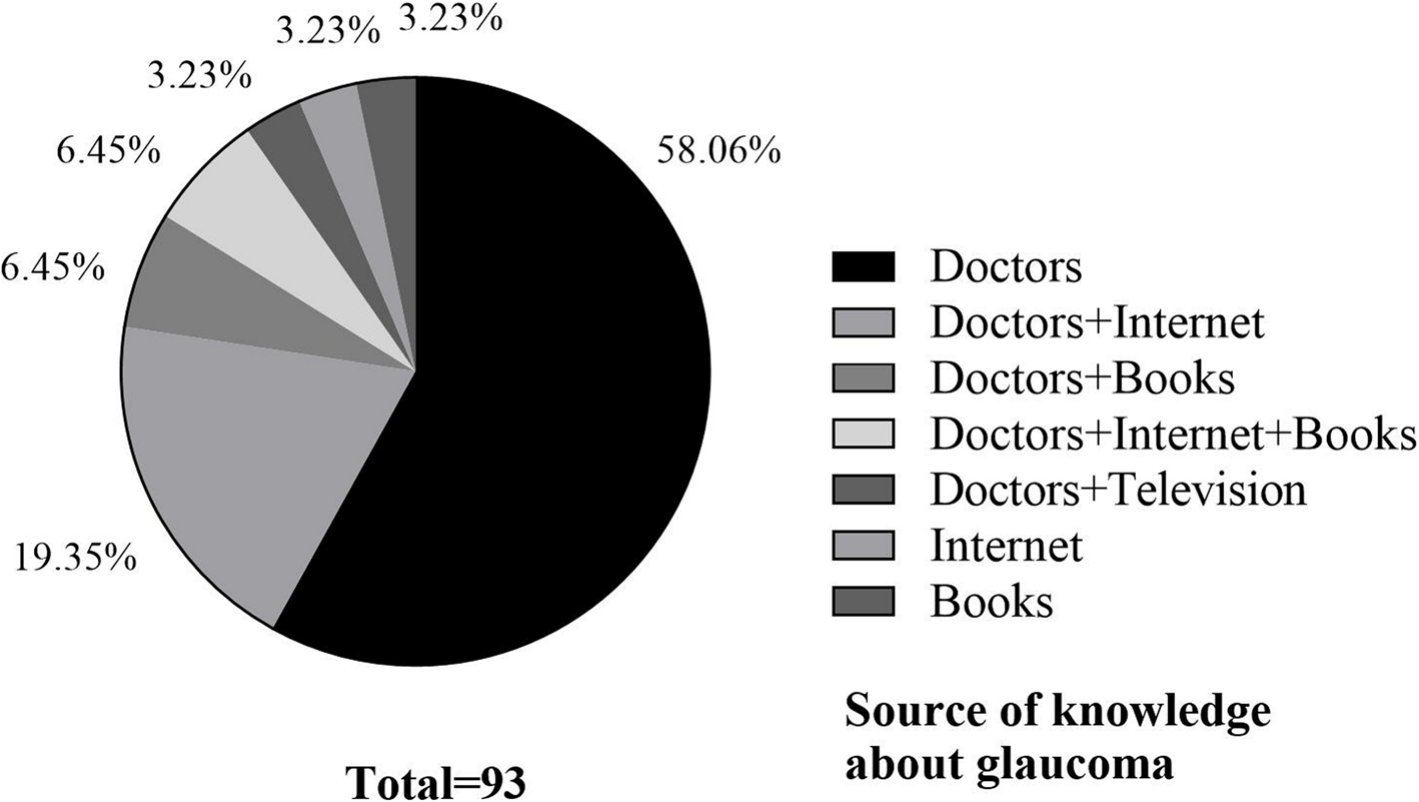
Source of knowledge about glaucoma

Figure 2 presented the sources of knowledge about glaucoma among patients in this study. The main source of knowledge for patients was doctors, followed by the internet. Eighty-seven (93.54%) got their knowledge from the doctors, 27 (29.03%) learned about their disease from the internet. Weak sources included media sources such as books and television.

### Knowledge of glaucoma and compliance to anti-glaucoma medications

In this study, 72 patients answered the questions regarding compliance to anti-glaucoma medications. We used the two questions “Can you use all the anti-glaucoma medications on time?” and “Have you ever stopped the medications on your own?" to assess the compliance to anti-glaucoma medications. 57 (79.17%) respondents could use all the anti-glaucoma medications on time, out of which 39 (54.17%) patients had good knowledge of glaucoma while 18 (25.0%) patients had poor knowledge of glaucoma (*P* = 0.001). 22 (30.56%) respondents used to stop the medications on their own, out of which only 7 (9.72%) patients had good knowledge of glaucoma while 15 (20.83%) patients had poor knowledge of glaucoma (*P* = 0.002) (Table 5). Moreover, among patients who underwent explaining the side effects (*P* = 0.001, *P* < 0.001) and who understood the side effects (*P* = 0.008, *P* = 0.001) and aim of anti-glaucoma medications (*P* = 0.001, *P* = 0.006), more patients used all the medications on time, and fewer patients stopped the medications on their own (Table 5). Thus, patients with good knowledge of glaucoma and knowing the purpose and side effects of anti-glaucoma medications had better compliance to anti-glaucoma medications.

**Table 5:** Association between knowledge level of glaucoma and the compliance to anti-glaucoma medications

**Variable**	**Use all the medications on time**		**χ**^**2**^	***P***	**Stop the medications on one’s own**		**χ**^**2**^	*P*
	**Yes (%)**	**No (%)**			**Yes (%)**	**No (%)**		
**Knowledge of glaucoma**								
Poor	25.00	16.67	11.46	0.001	20.83	20.83	9.16	0.002
Good	54.17	4.17			9.72	48.61		
**Do the anti-glaucoma medications have side effects?**								
Yes	31.94	0.00	7.13	0.008	1.39	30.56	10.94	0.001
No	47.22	20.83			29.17	38.89		
**Have the side effects of the medications been explained to you?**								
Yes	38.89	0.00	12.06	0.001	1.39	37.50	15.72	< 0.001
No	40.28	20.83			29.17	31.94		
**What is the purpose of the medications you use?**								
Satisfactory	70.83	9.72	11.29	0.001	18.06	62.50	7.45	0.006
Unsatisfactory	8.33	11.11			12.50	6.94		

### Knowledge of glaucoma and G-QoL

Table 6 demonstrated the median score of GLQ-15 in patients with good knowledge of glaucoma was 18 (16 ~ 25.5), while the median score of GLQ-15 in patients with poor knowledge of glaucoma was 23(17.5 ~ 36.5). As higher scores indicating poorer G-QoL, patients with good knowledge of glaucoma had lower scores of GLQ-15 (*P* = 0.025). The result indicated that patients with good knowledge of glaucoma have better G-QoL.

**Table 6:** Association between knowledge of glaucoma and scores of GLQ-15

Knowledge of glaucoma	GQL-15	Z	*P*
Poor	23 (17.5^~^36.5)	-2.235	0.025
Good	18 (16^~^25.5)		

## Discussion

China is the world's most populous country and may have the greatest number of people who are at risk of primary glaucoma. However, few data have been available regarding the knowledge of glaucoma, and associated factors among primary glaucoma patients in China.

In this cross-sectional hospital-based study of primary glaucoma patients in Kunming, an important central city in southwest China, the knowledge, attitude, and practices of glaucoma was assessed. In this study, 59.14% of patients were aware of glaucoma. Information about glaucoma was mainly obtained from doctors (93.54%). More importantly, our study showed that the age of the patient and the duration of the disease were related to the patient's knowledge of glaucoma. Furthermore, the compliance to anti-glaucoma medications and G-QoL were better in patients with good knowledge of glaucoma.

Various population-based studies have shown that knowledge of glaucoma is low in developed countries and worse in developing countries [5–9, 11, 28]. However, there are few existing data on the knowledge of glaucoma in Chinese glaucoma patients. Chen et al. [29] reported that 63% of patients knew their glaucoma type, and 38% of patients indicated visual field as a measure of progression in Shanghai. The result of our study was similar to that of Chen. Only 55 (59.14%) patients knew what glaucoma and normal IOP was (Table 3). 36 (38.71%) patients understood the reason for examining the visual field (Table 3). Based on the current data, glaucoma knowledge level was relatively low among primary glaucoma patients in Kunming, especially for key issues about the development and control of glaucoma such as IOP and visual field.

Previous studies have reported that age [7, 13, 23, 29–33], education [7, 13, 14, 23, 27, 29, 31, 34, 35], occupation [14, 23, 30], income [12], travel distance to hospital [36], duration of glaucoma [6, 34], medication [7], type of glaucoma [29] may influence the knowledge of glaucoma. The present study identified similar risk factors that are in line with previous reports with univariate logistic analysis. The univariate logistic analyses showed that age, education, occupation, income, travel distance to hospital, duration of glaucoma, medication, type of glaucoma were statistically significant with the knowledge of glaucoma (*P* < 0.05) (Table 4). After adjusting for potential confounders in the multivariate analysis, only younger age and glaucoma duration were positively associated with the knowledge level of glaucoma (Table 4). The discrepancies might be explained partly by differences in the study design, the study populations, and the statistical methods used. Interestingly, age was a controversial factor in different reports. Some studies showed that the knowledge of glaucoma was better among older individuals [7, 32] while some reports demonstrated younger people had better knowledge of glaucoma [23, 30, 31, 33]. Our results demonstrated patients in the younger age group were more likely to have good knowledge of glaucoma (Table 4). This is presumably due to better understanding of disease and exchanging knowledge through new communication facilities such as the internet among the younger generation.

On the other hand, this contention is supported by another key finding of the present study: a majority of the respondents acquired knowledge of glaucoma through their doctors and the internet played the second major role in gaining information regarding glaucoma among 93 patients (Fig. 2). We speculate that glaucoma patients with a longer duration of disease might have chances to contact their doctors more times and get more knowledge of glaucoma from doctors. The younger patients prefer to use the internet to gain better understanding of their disease. The finding shows that glaucoma patients in southwest China have limited access to information about their disease (Fig. 2). The publicity and popularization of glaucoma knowledge in the mass media such as television, newspaper, and books are lacking. Government and health policymakers should be aware that there is a pressing need to look into the strategies and approaches that could increase the public awareness and knowledge of glaucoma.

Another finding of this study is that patients with good knowledge of glaucoma were more likely to have better compliance to anti-glaucoma medications (Table 5). Glaucoma is a chronic and severe disease calling for consistent lifelong therapy. Reports indicate that patients’ knowledge about a disease has significant benefits on compliance to treatment [37, 38]. It became evident in a study performed by Altangerel et al. which revealed that a lack of adequate education about glaucoma might be more significantly associated with poor follow-up rates than a lack of access to care in those identified as glaucoma suspects in the USA [5]. Mansouri et al. showed that knowledge about glaucoma was positively associated with compliance in a study performed in Switzerland [4]. We observed a similar result in Chinese primary glaucoma patients. The patients with good knowledge of glaucoma were more likely to use all anti-glaucoma medications on time and most of the respondents with good knowledge level didn’t stop the medications by themselves (Table 5). We also found that the patients who underwent explaining the side effects, who understood the purpose of medication treatment had better compliance with medication. (Table 5). Surveys of glaucoma patients indicate that there is room for improvement in patient education; specifically, adequate knowledge regarding the side effects and purpose of anti-glaucoma medication treatment contributes greatly to the control and management of glaucoma for patients.

The ultimate goal in glaucoma management is the maintenance of QoL through the preservation of vision. In our study, a validated questionnaire GQL-15 was used to assess the relationship between knowledge of glaucoma and G-QoL (Table 6). Patients with good knowledge of glaucoma had lower scores of GLQ-15, which indicated that patients with good knowledge of glaucoma had better G-QoL (Table 6). Similar findings that the level of understanding about glaucoma was positively associated with QoL in patients in Shanghai were demonstrated in a previous study by Kong et al. [20]. Thus, imparting knowledge about glaucoma might help patients enhance their G-QoL.

Our study has several other limitations that should be acknowledged. First, participants were enrolled from a public hospital in a city in southwest China, and patients with unreliable examination results for glaucoma diagnosis were excluded from the present analyses. Thus, it could be affected by selection bias. Whether the findings could be extrapolated to the patients in other regions of China remains uncertain. Second, interviewer bias could not be eliminated as an individual's expression and style of explanation may affect the participant's response. Data were also based on self-reporting, which is subject to recall bias.

## Conclusions

In summary, our study provides relevant data on the knowledge of glaucoma and associated factors among primary glaucoma patients in Kunming, southwest China. Based on this study, the knowledge of disease in glaucoma patients is still low and inadequate. Improving knowledge of disease for patients through effective multiple means rather than relying only on ophthalmologists may be a veritable key step in combating blindness from glaucoma and enhancing patients’ QoL.

## Supplementary Information

**Additional file 1.**

## Data Availability

All data generated or analysed during this study are included within the article (and its Supplementary file S1).

## Abbreviations

POAG: Primary open angle glaucoma
JOAG: Juvenile open-angle glaucoma
PACG: Primary angle-closure glaucoma
BCVA: Best-corrected visual acuity
GQL-15: Glaucoma Quality of Life-15 questionnaire
QoL: Quality of life
G-QoL: Glaucoma-related quality of life
COR: Crude odds ratio
AOR: Adjusted odds ratio
CI: Confidence interval
